# Long noncoding RNAs to predict postoperative recurrence in bladder cancer and to develop a new molecular classification system

**DOI:** 10.1002/cam4.4443

**Published:** 2021-11-24

**Authors:** Zhiyong Li, Lijuan Jiang, Zhiling Zhang, Minhua Deng, Wensu Wei, Huancheng Tang, Shengjie Guo, Yunlin Ye, Kai Yao, Zhuowei Liu, Fangjian Zhou

**Affiliations:** ^1^ State Key Laboratory of Oncology in Southern China Guangzhou China; ^2^ Department of Urology Sun Yat‐sen University Cancer Center Guangzhou China; ^3^ Collaborative Innovation Center for Cancer Medicine Guangzhou China

**Keywords:** biomarker, bladder cancer, lncRNA, molecular subtypes, recurrence

## Abstract

**Background:**

Reliable molecular markers are much needed for early prediction of recurrence in muscle‐invasive bladder cancer (MIBC) patients. We aimed to build a long‐noncoding RNA (lncRNA) signature to improve recurrence prediction and lncRNA‐based molecular classification of MIBC.

**Methods:**

LncRNAs of 320 MIBC patients from the Cancer Genome Atlas (TCGA) database were analyzed, and a nomogram was established. A molecular classification system was created, and immunotherapy and chemotherapy response predictions, immune score analysis, immune infiltration analysis, and mutational data analysis were conducted. Survival analysis validation was also performed.

**Results:**

An eight‐lncRNA signature classifed the patients into high‐ and low‐risk subgroups, and these groups had significantly different (disease‐free survival) DFS. The ability of the eight‐lncRNA signature to make an accurate prognosis was tested using a validation dataset from our samples. The nomogram achieved a C‐index of 0.719 (95% CI, 0.674–0.764). Time‐dependent receiver operating characteristic curve (ROC) analysis indicated the superior prognostic accuracy of nomograms for DFS prediction (0.76, 95% CI, 0.697–0.807). Further, the four clusters (median DFS = 11.8, 15.3, 17.9, and 18.9 months, respectively) showed a high frequency of TTN (cluster 1), fibroblast growth factor receptor‐3 (cluster 2), TP53 (cluster 3), and TP53 mutations (cluster 4), respectively. They were enriched with M2 macrophages (cluster 1), CD8^+^ T cells (cluster 2), M0 macrophages (cluster 3), and M0 macrophages (cluster 4), respectively. Clusters 2 and 3 demonstrated potential sensitivity to immunotherapy and insensitivity to chemotherapy, whereas cluster 4 showed potential insensitivity to immunotherapy and sensitivity to chemotherapy.

**Conclusions:**

The eight‐lncRNA signature risk model may be a reliable prognostic signature for MIBC, which provides new insights into prediction of recurrence of MIBC. The model may help clinical decision and eventually benefit patients.

## Abstract 2

Reliable molecular markers are much needed for early prediction of recurrence in patients with muscle‐invasive bladder cancer (MIBC). It's the first time to build an lncRNA signature to improve recurrence prediction and for lncRNA‐based molecular classification of MIBC, which can be used to guide clinical treatment and provide precision therapy.

## 1 INTRODUCTION

Bladder cancer is one of the most frequently diagnosed cancers globally and the second most common cancer of the genitourinary tract.^1^, ^2^ Muscle‐invasive bladder cancer (MIBC) is associated with a 5‐year survival rate of 60% in case of patients with localized disease and <10% in cases with distant metastases.^3^ Radical cystectomy (RC) is the standard method of treatment. However, a “surgery only” study reported that the 5‐year recurrence‐free survival was 74%, 52%, and 36% in case of patients with pT2, pT3, and pT4, respectively.^4^ More than 20% patients with bladder cancer experience postoperative recurrence, leading to poor survival.^2^ Although the use of adjuvant chemotherapy after RC for patients with pT3/4 and/or (lymph node) LN‐positive disease without clinically detectable metastases is still under debate, it is effective for treating patients with MIBC with a high risk of recurrence.^5^, ^6^, ^7^ However, clinicopathological risk factors and TNM staging cannot adequately classify patients into high‐ or low risk of disease recurrence groups, and thus, they cannot indicate which patients are likely to benefit from postoperative chemotherapy. Consequently, there is an urgent need to identify novel, and reliable recurrence‐associated molecular biomarkers that allow for better prognostic stratification and can provide better guidance on therapy selection for patients with bladder cancer.

MIBC is a heterogeneous condition that is characterized by genomic instability and a high mutation rate.^8^ Bladder cancer can be stratified into different molecular subtypes for predicting outcome and treatment response; however, most previous studies have focused on the expression levels of mRNA to differentiate among molecular subtypes.^9^, ^10^ Long noncoding RNAs (lncRNAs) lack the protein‐coding function and are usually >200 bp in length.^4^, ^11^ Their aberrant expression has been closely associated with various types of cancers; moreover, lncRNAs have been experimentally validated to be involved in the etiology, pathogenesis, and progression of cancers.^12^ Furthermore, lncRNAs are a novel biomarker and therapeutic target for bladder cancer.^13^ For example, a recent study showed that as a novel regulator, lnc‐LBCS plays an important tumor suppressor role in bladder cancer stem cells’ self‐renewal and chemoresistance, contributing to weak tumorigenesis and enhanced chemosensitivity.^14^ Another study indicated that lncRNA DANCR induces bladder cancer lymph node metastasis and proliferation via an leucine‐rich pentatricopeptide repeat containing‐mediated mRNA stabilization mechanism.^15^ However, the potential of lncRNA‐based signatures and using lncRNAs for the molecular classification of bladder cancer has not been sufficiently explored.

In this study, we aimed to assess the ability of lncRNA expression profiles to predict disease recurrence; further, we attempted to use lncRNAs for the molecular classification of MIBC, which could guide clinical treatment in patients with MIBC.

## 2 METHODS

### 2.1 Patients with MIBC

We downloaded a TCGA–BLCA RNA sequencing dataset and corresponding clinical characteristics of patients from TCGA website (https://cancergenome.nih.gov/). In total, 430 BLCA tissues were identified, but the clinical characteristics of only 413 patients could be obtained. We then selected 320 patients with MIBC who either showed recurrence/progression or were disease free. Furthermore, mutational data pertaining to BLCA samples were downloaded from TCGA database.

### 2.2 Data processing

The downloaded BLCA data were normalized using the robust multichip average method for background correction, quantile normalization, and log_2_ transformation.^16^ The data were then annotated using the GENCODE (v26) GTF file. On using the DESeq2 R package, we identified 104 differentially expressed lncRNAs (DElncRNAs) with |logFC| >1 and adjusted *p* < 0.05 between patients with recurrence and non‐recurrence (Figure S1A). The samples were then randomly classified into training and validation groups at a 1:1 ratio. The LASSO Cox selection method was used to select the most appropriate number of lncRNAs to categorize the samples into those with recurrence/progression and those that were disease free.

### 2.3 Construction of a recurrence‐associated lncRNA signature and nomogram building

Univariate Cox regression survival analysis was used for selecting DElncRNAs associated with survival in bladder cancer. We constructed a disease‐free survival (DFS) risk‐score formula by including eight genes, weighted by their estimated regression coefficients in the univariable Cox regression analysis. Each patient was subjected to the risk‐score model and classified into a high‐ or low‐risk group using the median risk score of as the cut off point. A Kaplan–Meier curve and log‐rank test were used to assess DFS between the high‐ and low‐risk groups. Multivariate analysis with Cox regression proportional hazards regression were performed on lncRNA risk score, stage, age, invasion depth, grade, and gender. Hazard ratio (HR) and 95% confidence interval (CI) were also calculated. Furthermore, ROC analyses were performed to assess the sensitivity and specificity of recurrence prediction based on the lncRNA risk score (lncScore), stage, and invasion depth. In the log‐rank test, Cox regression analysis, and ROC analysis, *p* < 0.05 was considered statistically significant.

### 2.4 Cluster classification using lncRNAs

Using the differential expression profiles of lncRNAs of patients with and without recurrence, bladder cancer subtypes were obtained with unsupervised clustering analysis (using the R package “ConsensusClusterPlus”). After critically evaluating the obtained output (tracking plots, delta plots, and consensus cumulative distribution function [CDF] plots), a four‐cluster solution was found to be the most appropriate and informative. We then performed expression clustering analyses using the consensus partitioning around k‐medoids approach, with Pearson correlations, and 10,000 iterations with a 0.95 random fraction of lncRNAs in each iteration. Survival analyses were performed for the four bladder cancer subtypes.

### 2.5 Immunotherapy and chemotherapy response prediction, immune score analysis, immune infiltration analysis, and mutational data analysis

Tumor immune dysfunction and exclusion (TIDE) were used to predict immunotherapy response of the four clusters. To compute the TIDE scores, the TIDE web application (http://tide.dfci.harvard.edu/) was used. The R package “pRRophetic” was applied for to predict the chemotherapy responses of the four clusters.

The R package “ESTIMATE” was used to score the bladder cancer samples. The gene expression data of the samples were used to evaluate the stromal and immune scores of each sample through ESTIMATE; further, differences in scores were compared. The R package “maxstat” was used to determine the optimal cut off point for continuous variables, and the rank sum test was used to test the significance of survival between the high‐ and low‐risk groups. The relationships between score, stage, and clinical characteristics were assessed.

CIBERSORT was used to evaluate the different tumor‐infiltrating immune cells of bladder cancer samples of different subtypes. By inputting the expression data of bladder cancer samples, the proportions of 22 tumor‐infiltrating immune cells in each sample were obtained, and the samples with *p* < 0.05 were selected for subsequent analysis. We then determined the proportions of different tumor‐infiltrating immune cells subtypes. The relationship between the different tumor‐infiltrating immune cells ratios and survival within the four clusters and the median DFS of each cluster was assessed.

Mutational data pertaining to bladder cancer samples were downloaded from TCGA database, and the mutation load was calculated using this information. Based on the clinicopathological data, a mutation map of bladder cancer subtypes was constructed to study the types and clinical characteristics of gene mutations. The R package “maftools” and somatic signature were applied for analyzing the mutations and mapping the mutational spectrum and characteristics.

### 2.6 Statistical analysis

Statistical analyses were performed with the R statistical software (R Foundation for Statistical Computing). The primary endpoint for the survival analysis was disease‐free survival (DFS). DFS was defined as the date of radical cystectomy until the date of recurrence or death due to any cause. Patients who were lost to follow‐up were censored at the date of last contact. We used the Kaplan–Meier method to assess the statistical significance of differences between survival curves for patients in different lncRNA signature groups and molecular subtypes, with the log‐rank test.

## 3 RESULTS

### 3.1 Acquisition of lncRNA expression datasets

**FIGURE 1:**
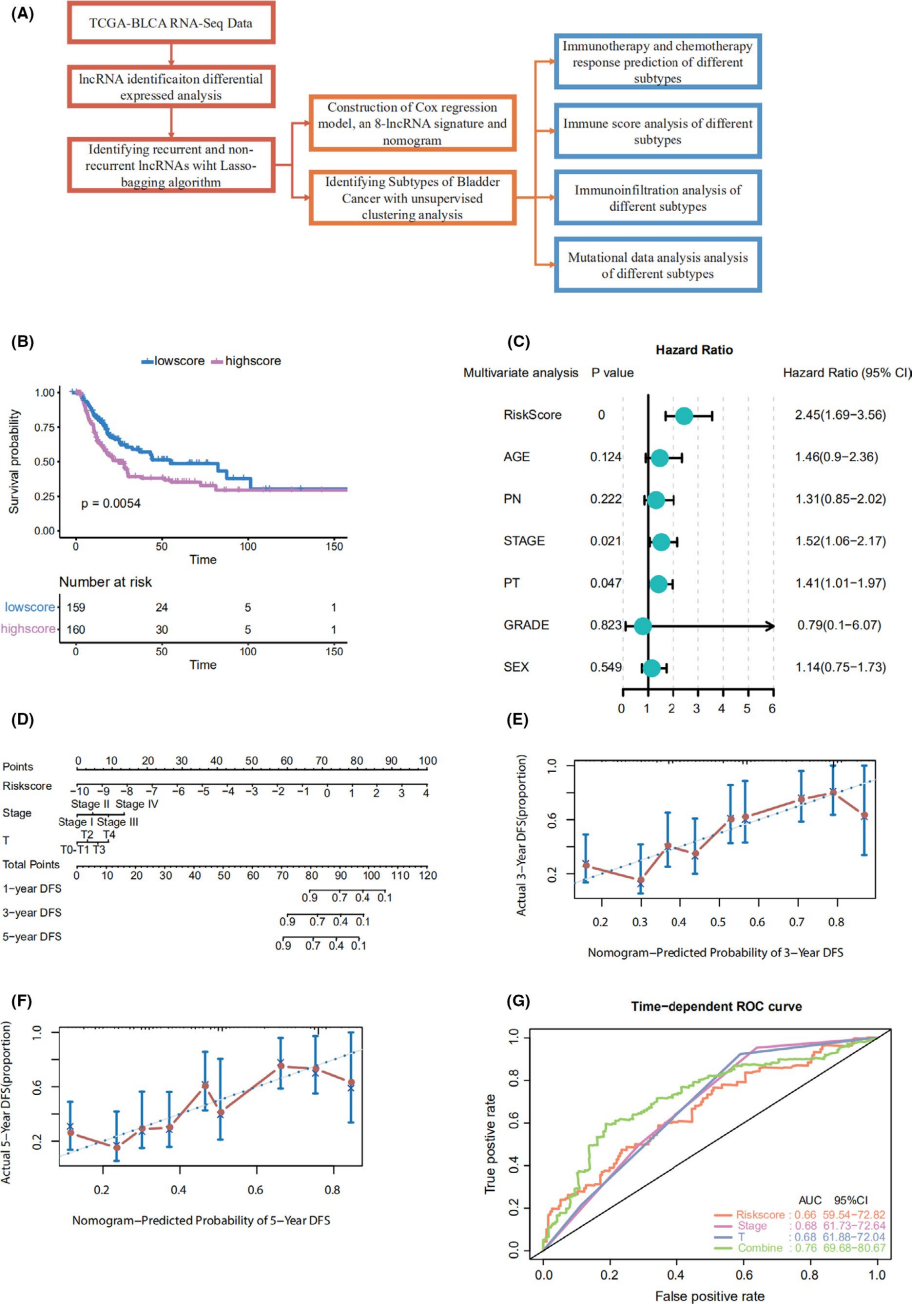
(A) Flowchart of study. (B) Kaplan–Meier curves of DFS based on the lncScore in MIBC patients. (C) Construction of a Cox model for DFS. (D) Nomogram to predict the DFS of MIBC patients. (E) Calibration curves of the nomogram to predict the 3‐year DFS. (F) Calibration curves of the nomogram to predict the 5‐year DFS. (G) Prediction of DFS by time‐dependent ROC analysis

Datasets and correlated clinical data were downloaded from TCGA database. In total, 320 patients with bladder cancer and follow‐up data were included (142 patients with recurrence and 178 with non‐recurrence). The flow chart is shown in Figure 1A. We compared the expression data pertaining to bladder cancer with the genome of the human v22 version of the GENCODE database, which led to the identification of 7648 lncRNAs. We then used the R package DESeq2 and applied the significance analysis of microarrays method with |logFC| >1 and *p* < 0.05; consequently, from the 7648 lncRNAs, 104 were found to be differentially expressed between patients with and without recurrence. We then randomly categorized the 320 patients with bladder cancer into training (*n* = 160) and validation sets (*n* = 160). The LASSO Cox selection method was applied and selected 86 differential expressed lncRNAs (DElncRNASs; Figure S1B). An ROC curve was constructed to evaluate the classification effect. The area under the ROC curve (AUC) was 1 for the training set and 0.792 for the validation set (Figure S1C,D). Therefore, these DElncRNAs were considered to be candidate recurrence‐associated lncRNAs.

### 3.2 Development and identification of the lncRNA‐based recurrence prediction model and nomogram building

Using the 86 DElncRNAs, we performed univariable Cox regression proportional hazards analysis. Eight lncRNAs related to prognosis were screened out (LINC01449, MGC39584, CTD‐2008P7.1, RP5‐907D15.4, RP11‐789C1.2, RP11‐44D19.1, CTC‐296K1.4, and AF015262.2; *p* < 0.05). A risk‐score formula was derived based on the expression of these eight lncRNAs for DFS prediction: (0.03589833) × LINC01449 + (0.002352983) × MGC39584 + (−3.231363e‐05) × CTD‐2008P7.1 + (0.001964683) × RP5‐907D15.4 + (−0.01417278) × RP11‐789C1.2 + (0.02019916) × RP11‐44D19.1 + (−0.000741376) × CTC‐296K1.4 + (0.01122571) × AF015262.2. We then calculated the eight‐lncRNA signature risk score for each patient with bladder cancer and ranked them according to their risk scores. The median risk score (0.023) was the cut off point used to classify the patients into high‐ (*n* = 160) and low‐risk (*n* = 160) groups. The patients in the low‐risk group showed significantly longer median DFS than those in the high‐risk group (log‐rank test *p* = 0.0054; Figure 1B). To further test the prediction value of the eight‑lncRNA signature in another cohort, 35 patients from our hospital were used as an external validation dataset. Patients in the validation cohorts were divided into a high‐risk group and a low‐risk group based on the median value. Patients with a high‐risk score had a shorter DFS than those with a low‐risk score in samples from our hospital (HR = 3.03, 95% CI 1.06–8.64, *p* = 0.039; Figure S2A).

Through a stepwise backward selection process based on the AUC value, lncScore, stage, and pT remained in the final Cox model for DFS (Figure 1C). To develop a clinically applicable tool that could provide individualized estimation of 1‐, 3‐, or 5‐year DFS, a nomogram was constructed on the basis of the final Cox model for DFS (Figure 1D). The nomogram achieved a C‐index of 0.719 (95% CI, 0.674–0.764), and the calibration plots showed good consistency between the actual DFS probabilities and the predicted DFS (Figure 1E,F). Time‐dependent ROC analysis also indicated the superior prognostic accuracy of the nomograms (AUC, 0.760; 95% CI, 0.697–0.807; Figure 1G). These findings validated the importance of the proposed nomograms for DFS prediction.

### 3.3 Consensus classification construction using lncRNAs

**FIGURE 2:**
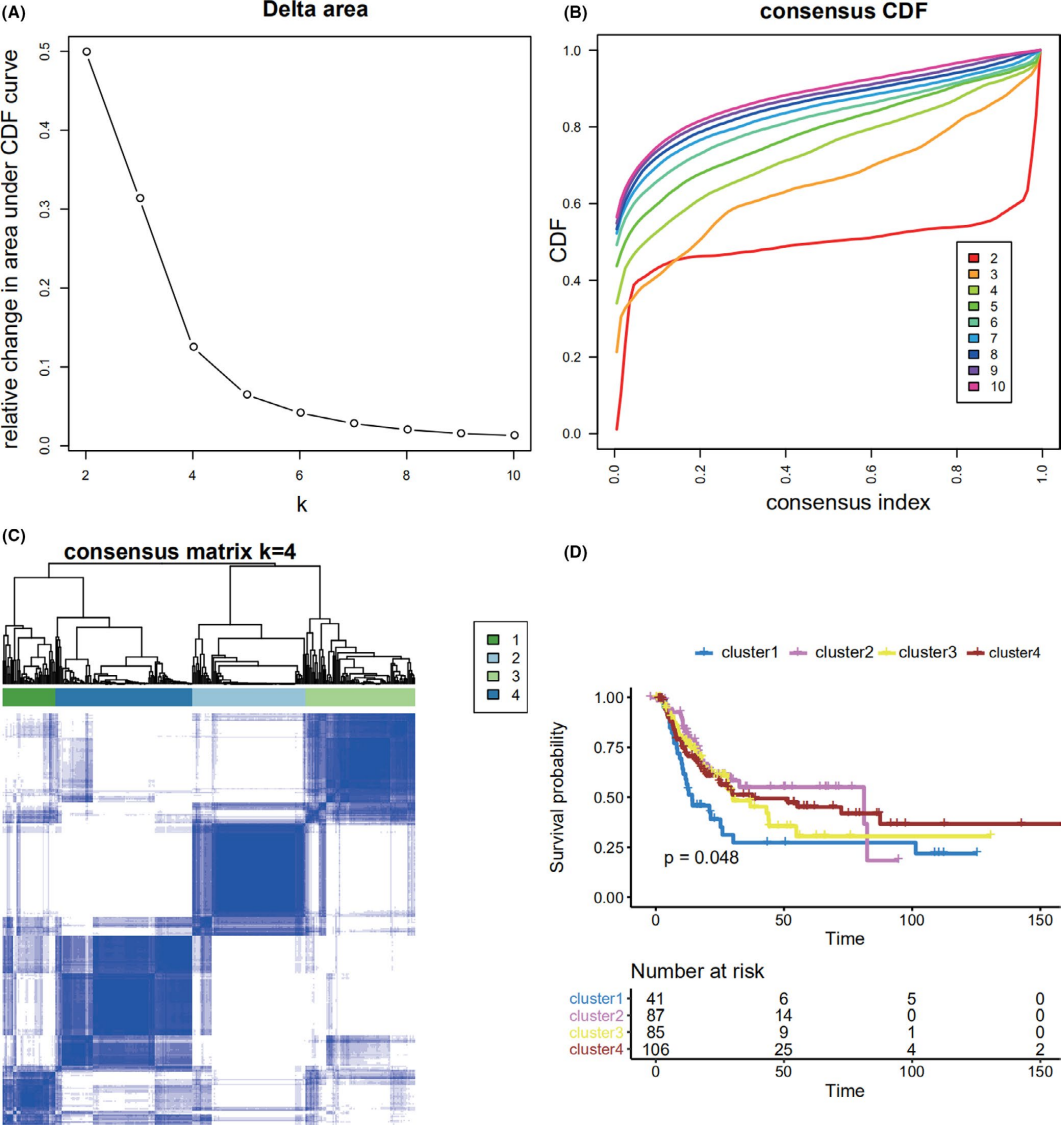
(A) Optimal *k*‐value selection graph for consistent clustering. (B) Consistent clustering CDF graph. (C) Sample clustering heatmap for *k* = 4. (D) Kaplan–Meier curves among the four subtypes of bladder cancer

Using the differential expression profiles of the 104 lncRNAs, unsupervised consensus clustering was used to derive a robust four‐cluster consensus solution (Figure 2A–C, Figure S2B–D). Survival analysis of the lncRNA‐based consensus clusters revealed significant survival differences among the four subtypes (Figure 2D).

The four clusters have different biological functions (Figure S3A–D). Cluster 1 was related to nicotine addiction, olfactory transduction, and taste transduction. Cluster 1 was related to nicotine addiction, olfactory transduction, and taste transduction. Cluster 2 was related to alpha‐linolenic acid metabolism, linolenic acid metabolism, and ribosome. Cluster 3 was related to linolenic acid metabolism, maturity onset diabetes of the young, and olfactory transduction. Cluster 4 was related to graft‐versus‐host disease, *Staphylococcus aureus* infection, and viral protein interaction with cytokine and cytokine receptor.

### 3.4 Prediction of immunotherapy and chemotherapy response for the new molecular classification system

**FIGURE 3:**
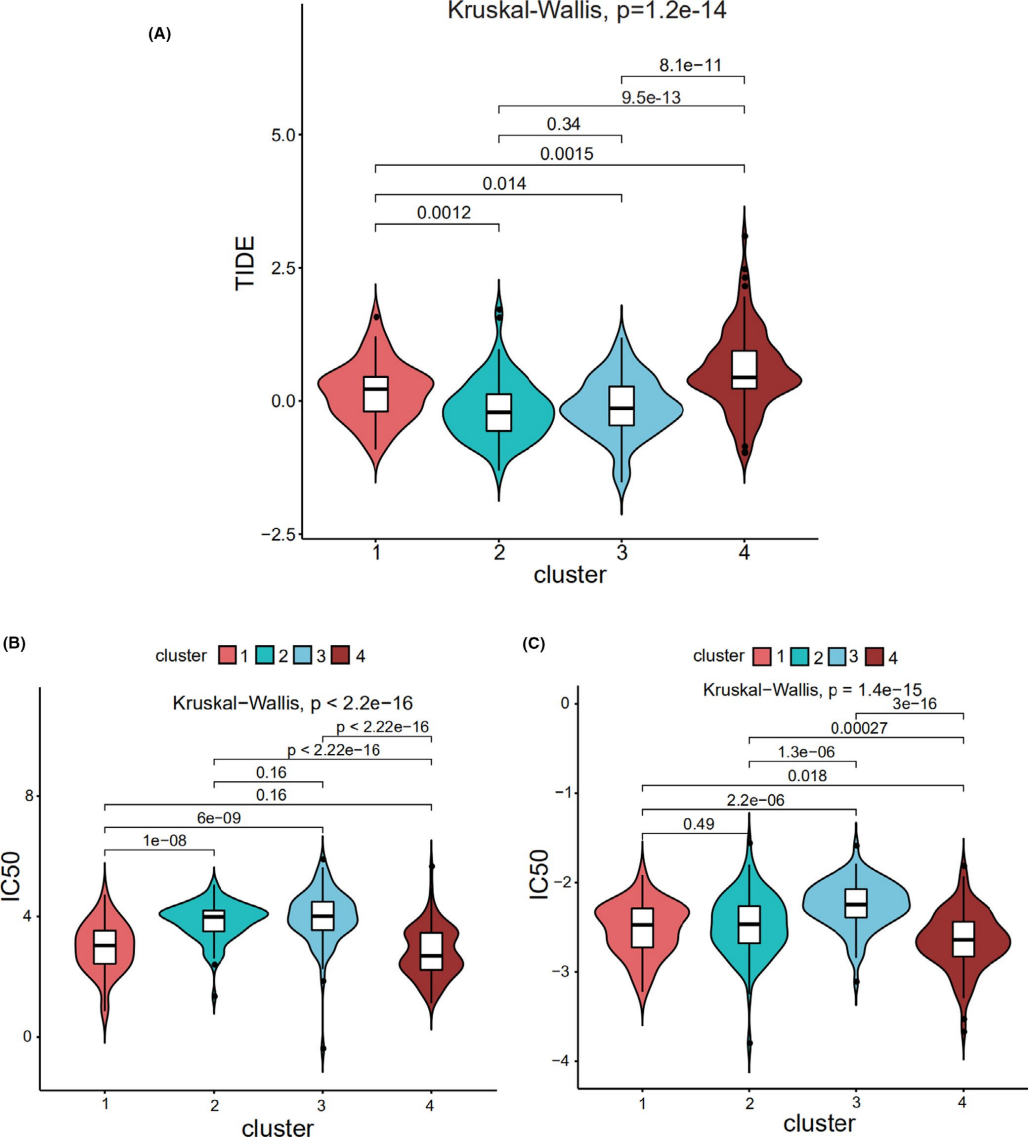
(A) Prediction of ICI therapy for four subtypes of bladder cancer. (B) Prediction of cisplatin for four subtypes of bladder cancer. (C) Prediction of gemcitabine for four subtypes of bladder cancer

TIDE was used to assess the clinical effects of immunotherapy in the four different subtypes of bladder cancer patients; significant differences were found in TIDE values among them (*p* = 1.2e^−14^; Figure 3A), which suggested that there were differences in the sensitivity of the four subtypes to immunotherapy. A high TIDE predictive score is associated with a poor effect of immune checkpoint suppression therapy. Cluster 4 showed a higher TIDE predictive score and thus appeared resistant to immunotherapy. In contrast, clusters 2 and 3 showed lower TIDE predictive scores and thus appeared sensitive to immunotherapy. Thus, depending on the subtype, we could determine when immunotherapy would be effective.

“pRRophetic” was used to predict the half maximal inhibitory concentration (IC50) values, which reflect the sensitivity of a sample to a drug, of the two most commonly used chemotherapy drugs (cisplatin and gemcitabine) for the four subtypes. The sensitivities of the four subtypes to cisplatin (Figure 3B) and gemcitabine (Figure 3C) were found to be significantly different. Cluster 4 was sensitive to cisplatin and gemcitabine, while clusters 2 and 3 were resistant to them.

### 3.5 Immune score analysis

**FIGURE 4:**
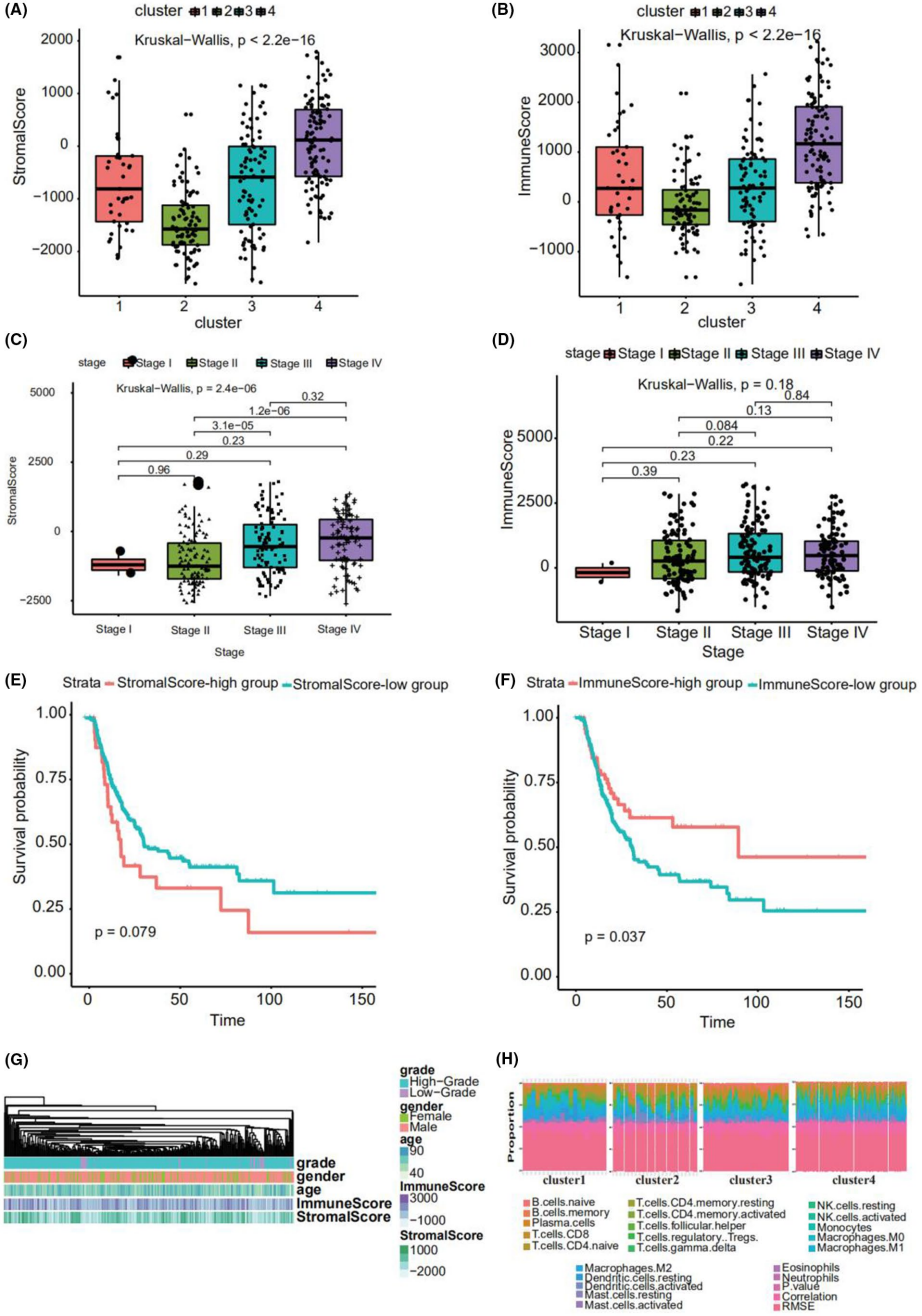
(A) Stromal scores of the four subtypes of bladder cancer. (B) Immune Scores of the four subtypes of bladder cancer. (C) Stromal scores of the different stages of bladder cancer. (D) Immune Scores of the different stages of bladder cancer. (E) Kaplan–Meier curves among high and low Stromal score groups. (F) Kaplan–Meier curves among high and low Immune score groups. (G) Heatmap of grade, gender, age, stromal score, and immune Score. (H) Proportions of tumor‐infiltrating immune cells among the four subtypes of bladder cancer

The gene expression data of bladder cancer samples were used to evaluate the stromal and immune scores, which represent tumor purity and can be used as indicators of tumor prognosis, using ESTIMATE. A significant difference was found between the scores among the four subtypes (Figure 4A,B). Cluster 2 showed a high stromal score and was thus associated with low tumor purity and poor prognosis. Cluster 4 showed a low stromal score and was accordingly associated with high tumor purity and favorable prognosis. Stromal score also increased with stage, consistent with high score, low purity, and poor prognosis (Figure 4C). Immune score also increased with an increase in stage (Figure 4D).

Using the “maxstat” R package to determine the best cut off point of continuous variables, patients were classified into high‐ and low stromal and immune score groups. A significant difference was found between these groups (*p* = 0.079 and 0.037, respectively; Figure 4E,F). The patients with a high stromal score showed worse prognosis. Figure 4G depicts a heatmap that reflects the relevant data.

### 3.6 Immune infiltration analysis

Using the expression (TPM) data, we predicted tumor‐infiltrating immune cells in different subtypes of bladder cancer with CIBERSORT; consequently, the proportion of 22 tumor‐infiltrating immune cells in each subtype of bladder cancer was obtained (Figure 4H). M2 Macrophages, CD8^+^ T cells, M0 macrophages, and M0 macrophages accounted for the largest proportion in clusters 1, 2, 3, and 4, respectively.

To understand the relationship between survival and the proportion of immune cells in the four clusters, we performed Cox regression analyses. In cluster 1, activated mast cells were related with survival. According to the median proportion of infiltrating cells, the samples were classified into high‐ and low‐expression groups, and significant differences in survival were found (Figure S4A). In cluster 2, resting mast cells and M1 and M0 macrophages were related to survival (Figure S4B–D). According to the median proportion of infiltrating cells, the samples were classified into high‐ and low‐risk groups, and significant differences in survival were only found for M0 macrophages. In cluster 3, resting memory CD4^+^ T cells, plasma cells, and resting mast cells were related to survival (Figure S4E–G). According to the median proportion of infiltrating cells, the samples were classified into high‐ and low‐expression groups, and significant differences in survival were found for resting memory CD4^+^ T cells and resting mast cells. Finally, in cluster 4, activated mast cells, M0 macrophages, naïve CD4^+^ T cells, and resting mast cells were related to survival (Figure S4H–K). According to the median proportion of infiltrating cells, the samples were classified into high‐ and low‐expression groups, and significant differences in survival were found for activated mast cells, M0 macrophages, and naïve CD4^+^ T cells.

### 3.7 Mutational data analysis

**FIGURE 5:**
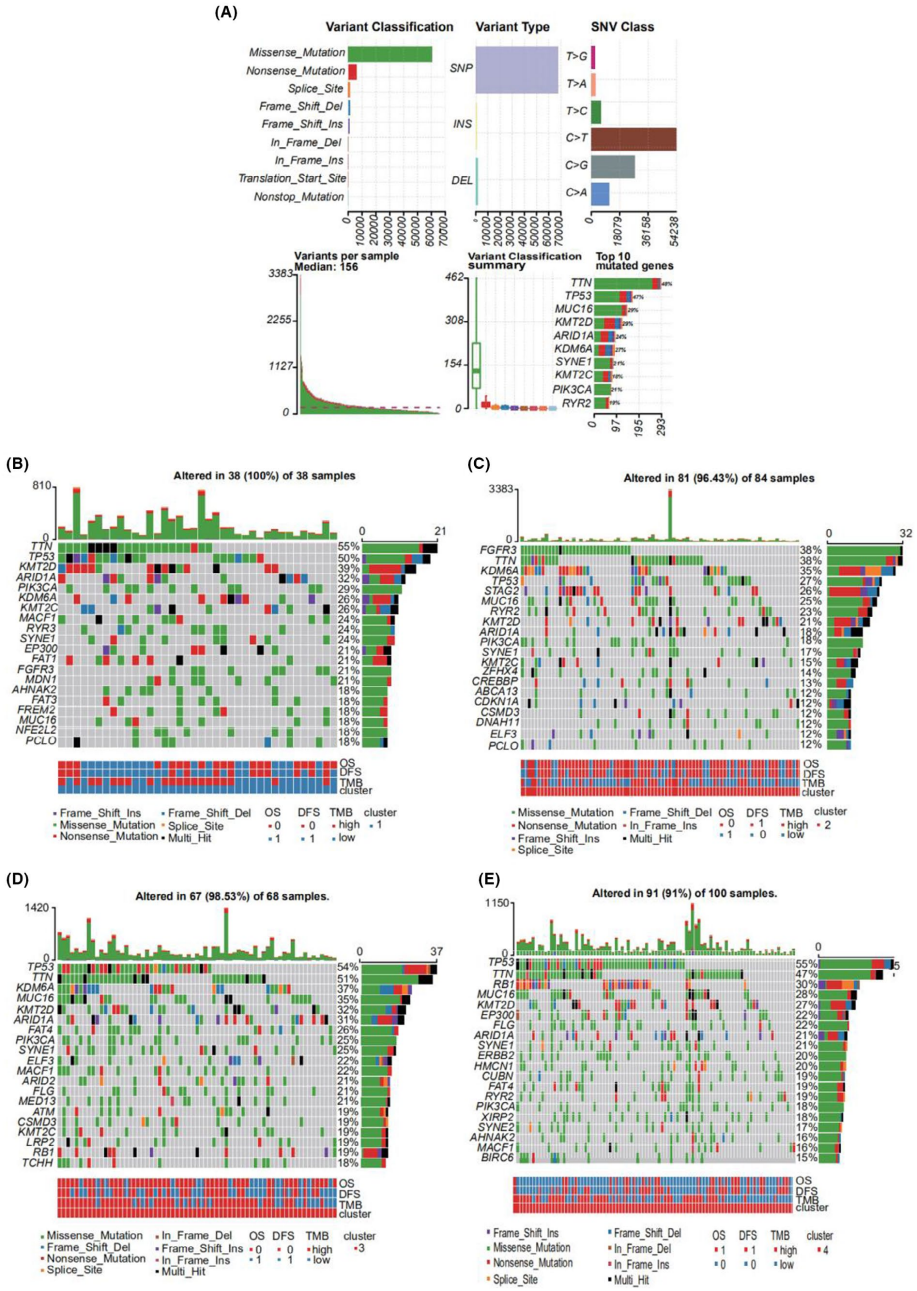
(A) Bladder cancer mutational profile. (B) Distribution of common gene mutations in bladder cancer cluster 1. (C) Distribution of common gene mutations in bladder cancer cluster 2. (D) Distribution of common gene mutations in bladder cancer cluster 3. (E) Distribution of common gene mutations in bladder cancer cluster 4

Using the mutational data from the TCGA database, we performed statistical analysis of the bladder cancer samples, including the type of mutation annotation, proportion of different types of base changes, and the top 10 mutated genes (Figure 5A). Missense mutations were the main type of mutation in bladder cancer; single nucleotide polymorphisms were the main source of mutation, followed by deletion (DEL) and insertion (INS). C>T was the most common single nucleotide polymorphism. TTN, TP53, and MUC16 were among the top 10 genes with highest incidences of mutations. The distribution, mutation annotation, survival status (overall survival [OS], DFS), and tumor mutation load of the four BLCA subtypes (clusters 1–4) are shown in Figure 5B–E. TTN was the gene with the highest mutation rate in cluster 1, fibroblast growth factor receptor (FGFR)‐3 had the highest mutation rate in cluster 2, and TP53 had the highest mutation rate in clusters 3 and 4. As evident from Figure S5A–D, the frequency of mutant genes (e.g., TP53 and KDM6A) was different in the four subtypes.

**FIGURE 6:**
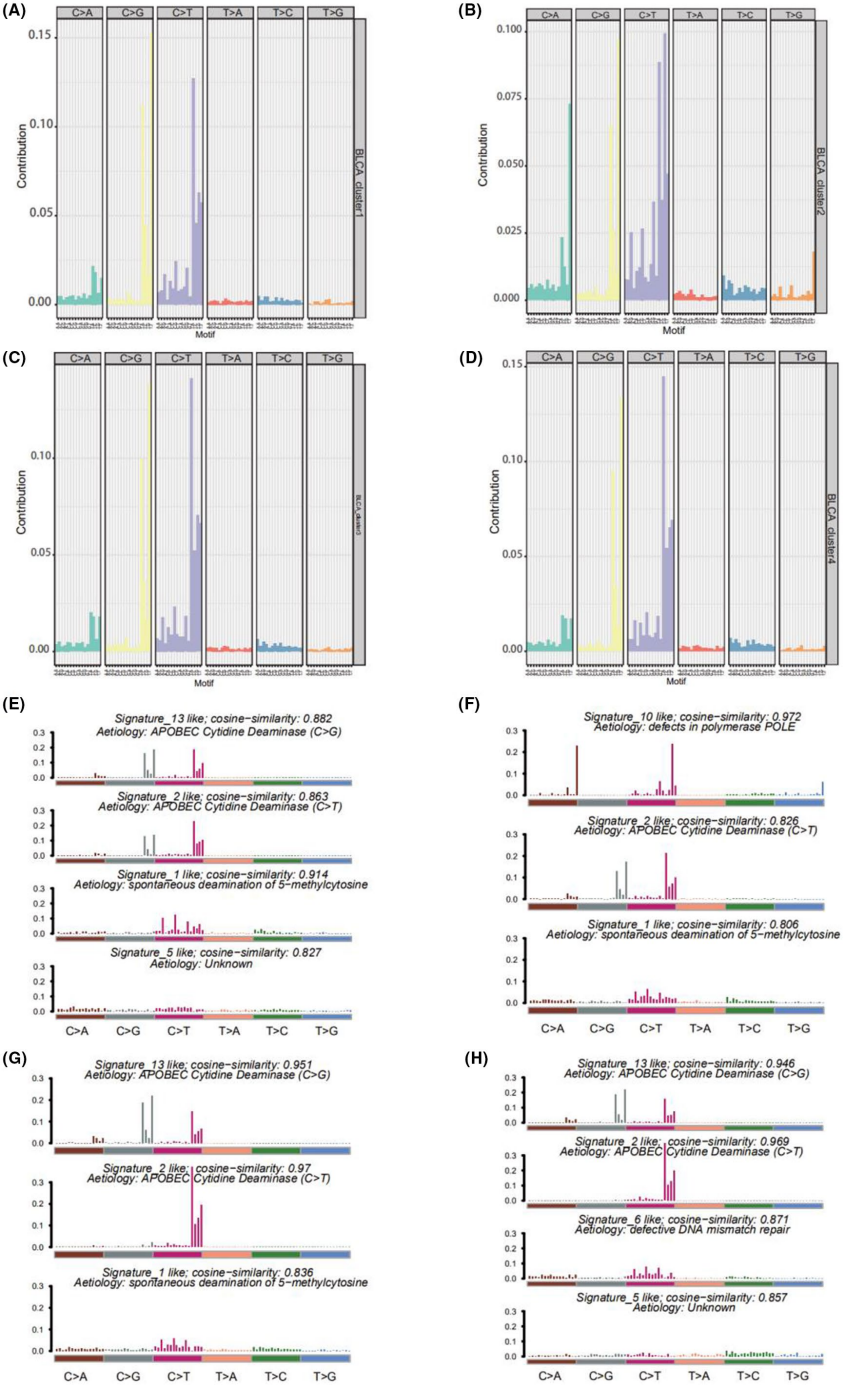
(A) Similarity between mutation characteristics of bladder cancer cluster 1 and cosmic mutation signature. (B) Similarity between mutation characteristics of bladder cancer cluster 2 and cosmic mutation signature. (C) Similarity between mutation characteristics of bladder cancer cluster 3 and cosmic mutation signature. (D) Similarity between mutation characteristics of bladder cancer cluster 4 and cosmic mutation signature. (E) Frequency distribution of 96 mutation types in bladder cancer cluster 1. (F) Frequency distribution of 96 mutation types in bladder cancer cluster 2. (G) Frequency distribution of 96 mutation types in bladder cancer cluster 3. (H) Frequency distribution of 96 mutation types in bladder cancer cluster 4

Based on the mutation site of each sample, we considered the bases at the 1‐bp position upstream and downstream of the mutation site, divided the mutations sites into 96 types, and counted the frequency of the 96 mutation types in tumor samples belonging to the four subtypes (Figure 6A–D).

To determine the relationship between the mutation frequency distribution and signatures collected from COSMIC, we performed nonnegative matrix decomposition on the frequency matrix with rows as samples and columns with the 96 mutation types to extract 2 and 3 somatic point mutation characteristics. The similarity analysis of the extracted features and collected signatures revealed that the BLCA mutation spectrum of the bladder cancer subtype 1 (cluster 1) was mainly related to signatures 13, 2, 1, 10, and 5; subtype 2 (cluster 2) was mainly related to signatures 10, 2, and 1; subtype 3 (cluster 3) was mainly related to signatures 13, 2, and 1; and subtype 4 (cluster 4) was mainly related to signatures 13, 2, 6, and 5 (Figure 6E–H). Signatures 13 and 2 were mainly related to adenosine deaminase, 1 was mainly related to the spontaneous deamination reaction of 5‐methylcytosine, 10 was mainly related to polymerase defects, and 6 was mainly related to DNA mismatch repair.

## 4 DISCUSSION

In this study, we developed a novel tool based on eight lncRNAs to improve the prediction of recurrence in patients with MIBC. The tool effectively stratified the patients into groups with a low‐ or high risk of recurrence. Furthermore, based on the differential expression of lncRNAs between patients with and without recurrence, four clusters with different DFS and molecular features were identified. To the best of our knowledge, this is the first study to demonstrate the clinical utility of an lncRNA signature for the postoperative prediction of recurrence in patients with MIBC and to establish an lncRNA‐associated cluster with molecular features.

In patients with MIBC, RC, and pelvic lymph node dissection are the standard methods of treatment.^2^ The effectiveness of adjuvant chemotherapy after RC for patients with pT3/4 and/or lymph node‐positive (N^+^) disease is still under debate.^17^ Local recurrence takes place in soft tissues of the original surgical site or in lymph nodes. Cystectomy is associated with 5%–15% probability of pelvic recurrence, which usually occurs during the first 24 months (most often within 6–18 months) after surgery.^18^ Distant recurrence is observed in up to 50% of patients with MIBC treated using RC.^2^ Most importantly, patients generally shows poor prognosis in post‐recurrence.^19^ Even with treatment, median survival ranges between 4 and 8 months after diagnosis. Thus, it is vital to identify high‐risk relapse patients and treat them as early as possible. Pertinent risk factors reportedly include pathological stage, positive lymph nodes, positive margins, extent of lymph node dissection, and perioperative chemotherapy without any molecular biological features.^20^ However, these factors cannot effectively predict the risk of postoperative recurrence in patients with bladder cancer. Although the TNM staging system and clinical factors are widely used to predict prognoses and guide treatment decisions in such patients, clinical factors are associated with some critical limitations because of the molecular heterogeneity of bladder cancer. Thus, we herein aimed to develop a molecular classification system that used lncScore to categorize samples into subsets with distinct outcomes, facilitating the development of treatment strategies and avoiding of overtreatment or undertreatment. Moreover, we developed a nomogram for the individualized assessment of 1‐, 3‐, and 5‐year DFS probabilities in patients with MIBC after RC. Our findings suggest that lncScore and recurrence‐associated nomogram can be used for predicting the risk of recurrence after RC and for guiding the use of adjuvant chemotherapy to reduce the risk of recurrence.

It has recently been reported that the expression patterns of functional lncRNAs are associated with human cancers.^21^, ^22^ These lncRNAs have been implicated in various tumorigenesis processes, including proliferation, invasion, and apoptosis.^23^, ^24^, ^25^ Some lncRNA‐based signatures have been used to predict the risk of cancer progression in patients with renal cell carcinoma and colon cancer.^26^, ^27^ Moreover, prognostic lncRNAs have previously been reported in bladder cancer; four lncRNAs—AC145124.1, AC010168.2, MIR200CHG, and AC098613.1—were reported to form a signature to predict survival in BLCA.^28^ Another study reported that a 12‐lncRNA‐based classifier was related to recurrence in all patients with bladder cancer.^29^ As for prediction of recurrence, the article's AUC value of lncRNA signature combining stage was 0.739, while AUC value of our nomogram was 0.760. Their lncRNA signature consist of 12 lncRNAs. Our lncRNA signature consist of 8 lncRNAs, with fewer numbers and similar prediction efficiency. As for novel approaches to monitor the recurrence and risk stratification of bladder cancer, a recent study showed that urine tumor DNA methylation had a high sensitivity of 89.5% to detect recurrence, achieving a great improvement in sensitivity over urine cytology and FISH.^30^ Furthermore, another study found that the urine‐based DNA methylation with a five‐marker stratification model identified high‐risk NMIBC and MIBC with 90.5% sensitivity and 86.8% specificity, representing a highly sensitive and specific approach for bladder cancer risk stratification.^31^ However, such studies have been limited by the small number of screened lncRNAs and the lack of prediction of recurrence risk in MIBC cases. These studies have chiefly focused on both non‐muscle invasive bladder cancer and MIBC. In this study, for the first time, we successfully identified an eight‐lncRNA signature to predict the risk of recurrence in patients with MIBC. Among the eight lncRNAs, MGC39584 may play a key role in lung squamous cell carcinoma.^32^ Furthermore, DNA hypermethylation of the promoter region of GJC1 (CTC‐296K1.4), encoding connexin 45, is an important mechanism in silencing gene expression in colorectal cancer.^33^ Unfortunately, the other novel lncRNAs have not yet been investigated in cancer.

Molecular subtyping plays a pivotal role in the study of diseases and for developing personalized therapeutics. The molecular characterization of MIBC by transcriptome profiling has revealed a range of subtypes with distinct clinicopathological characteristics, prognosis, and response to therapeutic regimens. Significant endeavors have been made in the molecular subtyping of MIBC to guide clinical treatment.^34^, ^35^, ^36^ However, previous studies have mainly focused on mRNA‐based molecular subtyping; it is noteworthy that mRNA transcripts only represent 1%–2% of the transcriptome, which is primarily dominated by ribosomal RNAs and ncRNAs.^21^ Thus, we herein selected a list of highly differentially expressed lncRNAs for consensus clustering and identified four clusters with different DFS and molecular features. We found a biologically distinct MIBC subgroup with potential clinical utility. Cluster 1 showed a high frequency of TTN mutation and enriched M2 macrophages. Cluster 2 demonstrated potential sensitivity to immunotherapy and insensitivity to chemotherapy, with a high frequency of FGFR‐3 mutations and enriched CD8^+^ T cells. The high frequency of FGFR‐3 mutations suggested that cluster 2 may respond to FGFR inhibitors. Novel FGFR inhibitors have been reported to clinically benefit 20% of patients with MIBC that have tumors harboring mutations or translocations in the tyrosine kinase receptor FGFR‐3 and 40% of those having tumors overexpressing FGFR‐3. Cluster 3 demonstrated potential sensitivity to immunotherapy and insensitivity to chemotherapy, with a high frequency of TP53 mutations and enriched M0 macrophages. Cluster 4 demonstrated potential insensitivity to immunotherapy and sensitivity to chemotherapy, with a high frequency of TP53 mutations and enriched M0 macrophages. PD‐1–PD‐L1 immune checkpoint blockade is becoming standard in patients with locally advanced or metastatic urothelial cancer who relapse after cisplatin‐based chemotherapy or are considered cisplatin ineligible (objective response rate = 20%).

This study has some limitations. First, some clinical information was missing (details pertaining to, for example, neoadjuvant and adjuvant treatment). Second, the retrospective nature of this study made it susceptible to inherent biases. Finally, because of the limited sample size, we could not use our lncRNA signature and nomogram to guide clinical treatment.

The eight‐lncRNA signature risk model may be a reliable prognostic signature for MIBC, which provides new insights into prediction of recurrence of MIBC. The model may help clinical decision and eventually benefit patients. However, large‐scale, multi‐center, and prospective studies are necessary to confirm our results before the eight‐lncRNA model‐based signature can be applied in the clinic.

## 5 ETHICS APPROVAL AND CONSENT TO PARTICIPATE

Not applicable.

## CONFLICT OF INTERESTS

The authors declare that they have no conflict of interest.

## AUTHOR CONTRIBUTIONS

ZYL, FJZ, and ZWL designed the study. KY, LJJ, ZLZ, MHD, WSW, and HCT collected and confirmed the data. SJG and YLY analyzed the data. ZYL and LJJ wrote the manuscript. All authors approved the final version of the manuscript.

## DISCLOSURE

The authors have no conflict of interest to declare.

## CONSENT FOR PUBLICATION

All listed authors have actively participated in the study and have read and approved the submitted manuscript.

## Supporting information

Supplementary Material

## Data Availability

RNA‐sequencing (RNA‐seq) and somatic mutation data of the TCGA database.
