# Supplementary material for: Long noncoding RNAs to predict postoperative recurrence in bladder cancer and to develop a new molecular classification system
Source: Cancer Med. 2021 Nov 24;11(2):539–52. doi: 10.1002/cam4.4443 (PMC8729057; doi:10.1002/cam4.4443)
Supplement: Supplementary file 1 — Supplementary Material [file CAM4-11-539-s001.pdf]

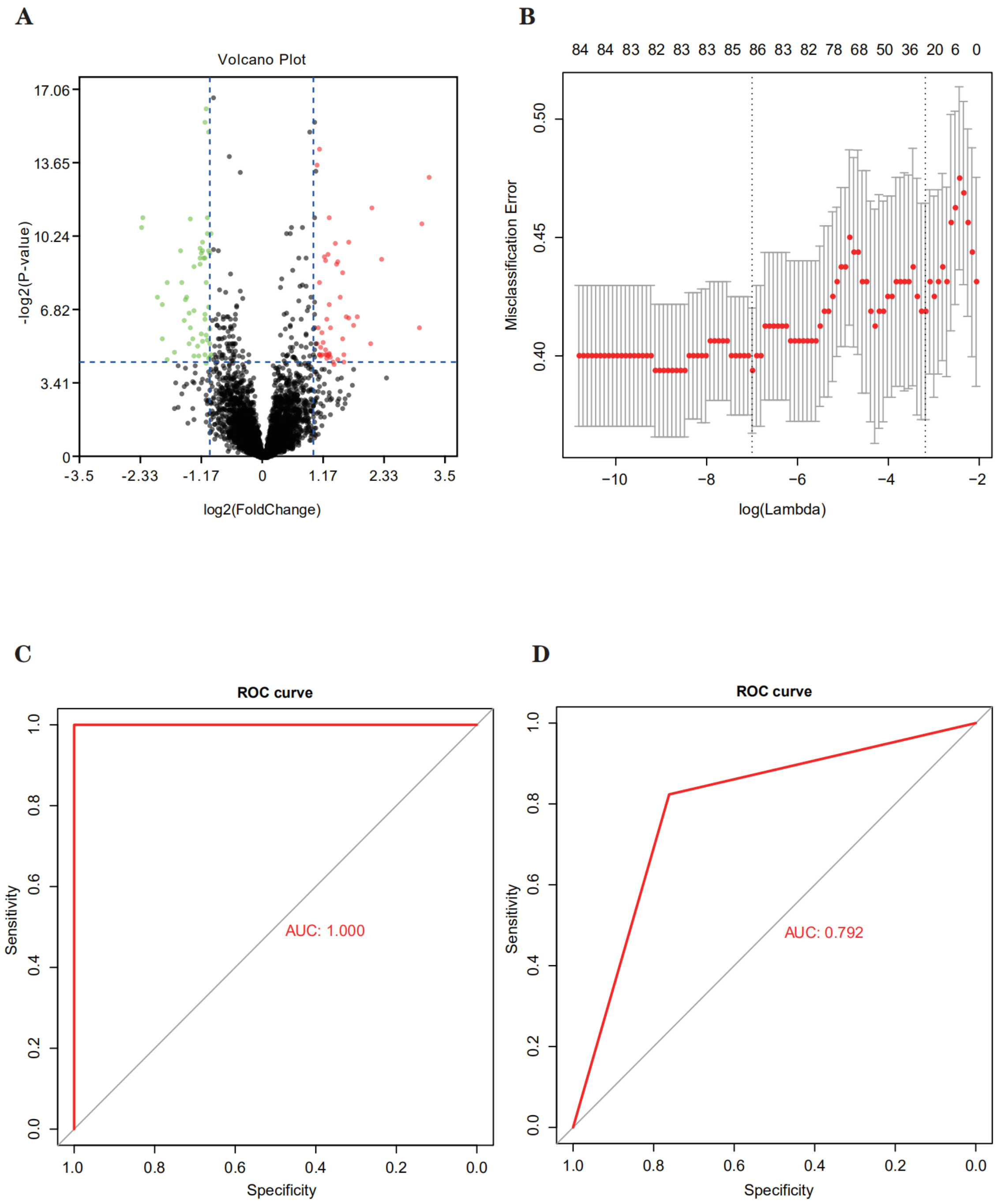

Figure S1A. Volcano plot of differentially expressed lncRNAs; Figure S1B. Classification feature number and error rate graph; Figure S1C. ROC curve of training set group; Figure S1D. ROC curve of test set group

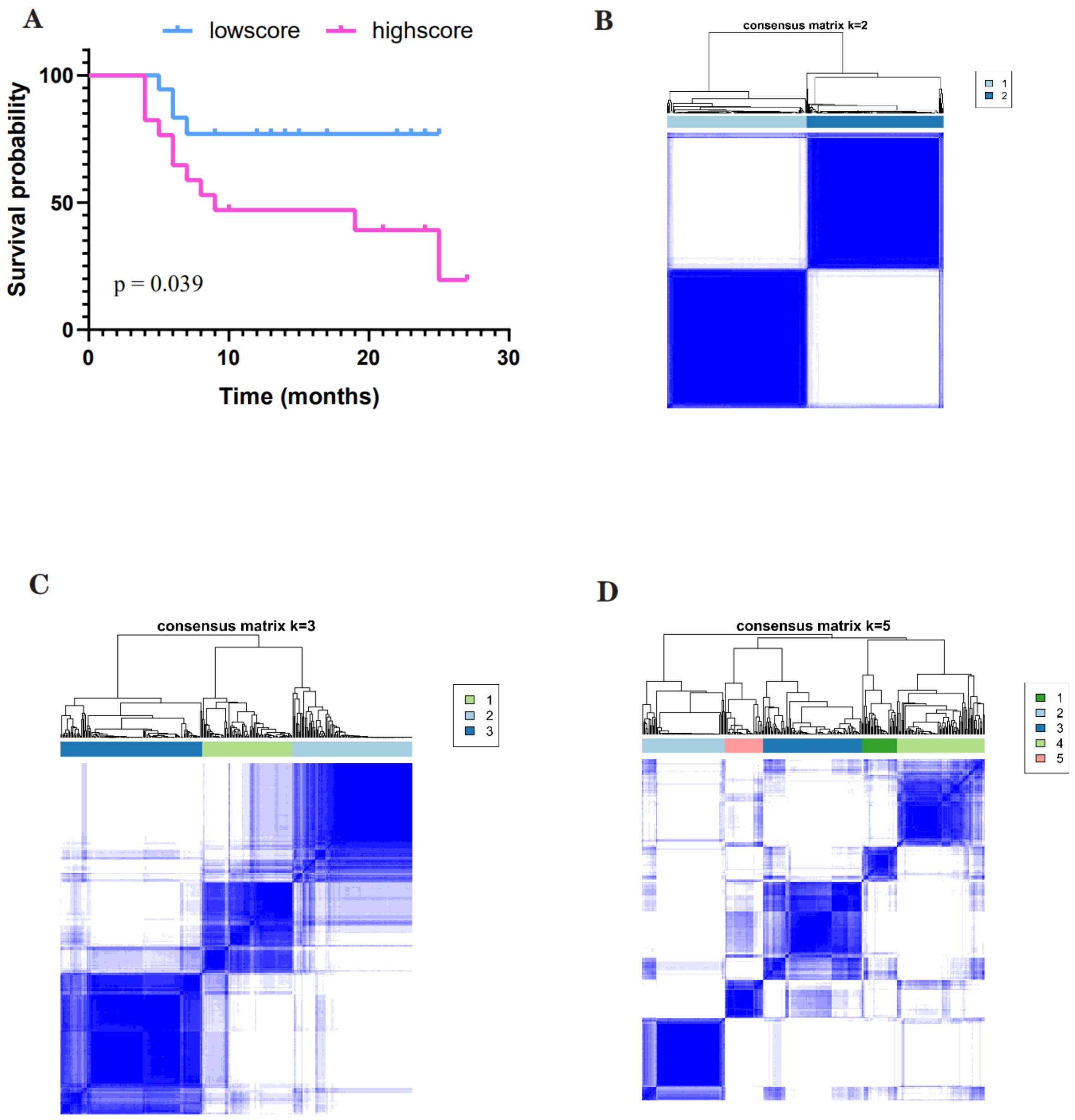

Figure S2A. Validation of the eight-lncRNA signature for prognosis in an independent dataset

Figure S2B. Sample clustering heatmap for k = 2

Figure S2C. Sample clustering heatmap for k = 4

Figure S2D. Sample clustering heatmap for k = 5

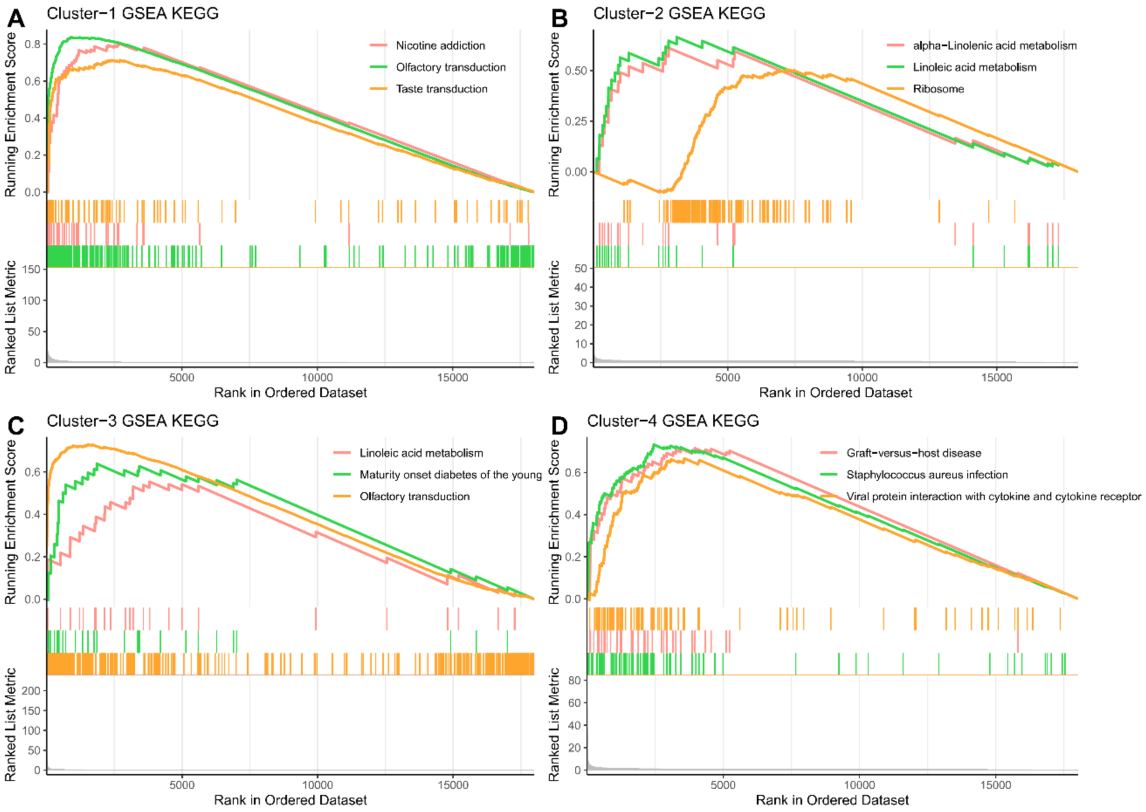

Figure S3A. Biological functions of the cluster 1

Figure S3B. Biological functions of the cluster 2

Figure S3C. Biological functions of the cluster 3

Figure S3D. Biological functions of the cluster 4

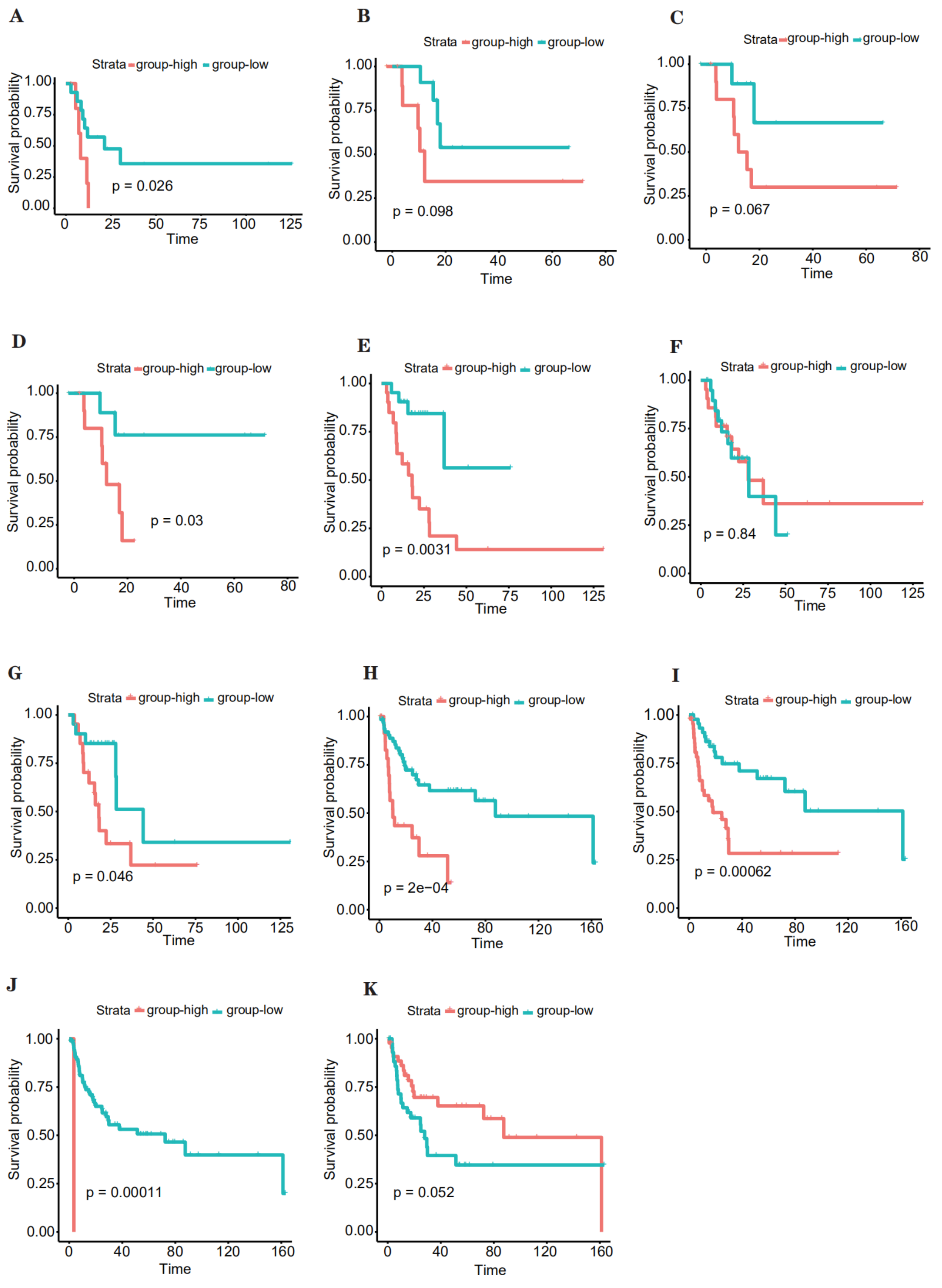

Figure S4A. Kaplan–Meier curves between high- and low-expression groups of mast cells in cluster 1; Figure S4B. Kaplan–Meier curves between high- and low-expression groups of mast cells in cluster 2; Figure S4C. Kaplan–Meier curves between high- and low-expression groups of M1 macrophages in cluster 2; Figure S4D. Kaplan–Meier curves between high- and low-expression groups of M0 macrophages in cluster 2;

Figure S4E. Kaplan–Meier curves between high- and low-expression groups of resting memory CD4+ T cells in cluster 3; Figure S4F. Kaplan–Meier curves between high- and low-expression groups of plasma cells in cluster 3; Figure S4G. Kaplan–Meier curves between high- and low-expression groups of resting mast cells in cluster 3; Figure S4H. Kaplan–Meier curves between high- and low-expression groups of activated mast cells in cluster 4; Figure S4I. Kaplan–Meier curves between high- and low-expression groups of M0 macrophages in cluster 4; Figure S4J. Kaplan–Meier curves between high- and low-expression groups of naïve CD4+ T cells in cluster 4; Figure S4K. Kaplan–Meier curves between high- and low-expression groups of resting mast cells in cluster 4

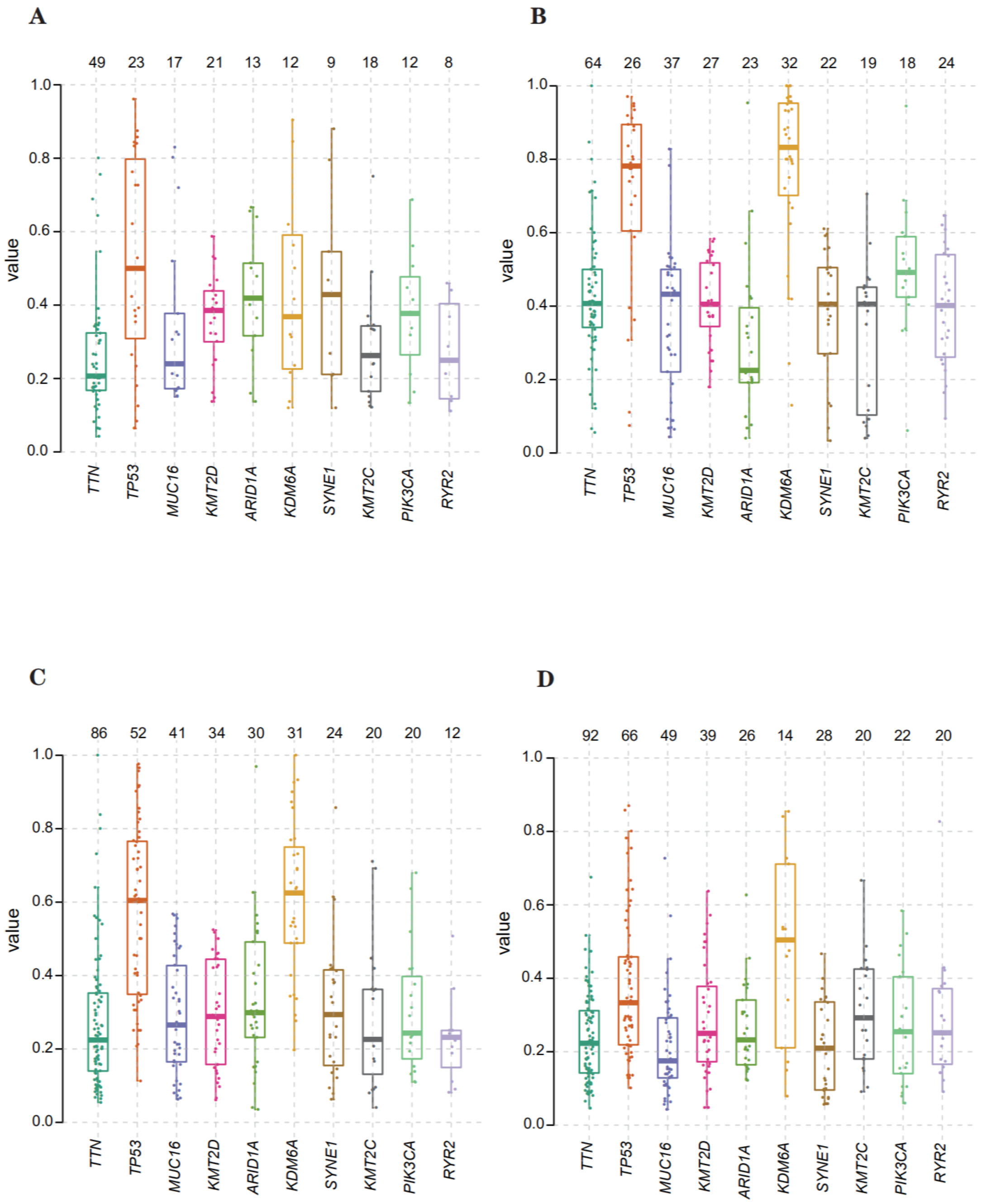

Figure S5A. Frequency distribution of common gene mutations in bladder cancer cluster 1; Figure S5B. Frequency distribution of common gene mutations in bladder cancer cluster 2; Figure S5C. Frequency distribution of common gene mutations in bladder cancer cluster 3; Figure S5D. Frequency distribution of common gene mutations in bladder cancer cluster 4
